# Non-invasive assessment of left atrial activation using lead V8 during Bachmann bundle pacing

**DOI:** 10.1093/europace/euag276

**Published:** 2026-09-25

**Authors:** Simon Mulier, Arno Fennema, Johan van Koll, Masih Mafi-Rad, Vidal Essebag, Atul Verma, Dominik Linz, Justin Luermans, Kevin Vernooy, Jacqueline Joza

**Affiliations:** Department of Cardiology, Cardiovascular Research Institute Maastricht (CARIM), Maastricht, The Netherlands; Department of Cardiology, Cardiovascular Research Institute Maastricht (CARIM), Maastricht, The Netherlands; Department of Cardiology, Cardiovascular Research Institute Maastricht (CARIM), Maastricht, The Netherlands; Department of Cardiology, Cardiovascular Research Institute Maastricht (CARIM), Maastricht, The Netherlands; Department of Medicine, McGill University Health Center, Montréal, Québec, Canada; Department of Medicine, McGill University Health Center, Montréal, Québec, Canada; Department of Cardiology, Cardiovascular Research Institute Maastricht (CARIM), Maastricht, The Netherlands; Department of Cardiology, Cardiovascular Research Institute Maastricht (CARIM), Maastricht, The Netherlands; Department of Cardiology, Cardiovascular Research Institute Maastricht (CARIM), Maastricht, The Netherlands; Department of Medicine, McGill University Health Center, Montréal, Québec, Canada

**Keywords:** Bachmann bundle pacing, Interatrial synchrony, Left atrial activation, Lead V8, Negative derivative

## Abstract

**Aims:**

Bachmann bundle pacing (BBP) improves atrial synchrony by advancing LA activation, but quantifying inter-atrial synchronization remains challenging. This study evaluated whether lead V8 reflects posterolateral LA activation time and whether lead V8 can be used to assess the effect of BBP on LA activation.

**Methods and results:**

Consecutive patients undergoing an electrophysiology study and ablation (Cohort 1) or BBP lead implantation (Cohort 2) were prospectively enrolled. Surface ECGs were recorded with the V5 electrode repositioned to the V8 location (2 cm below the inferior left scapula). LA activation was determined using the steepest negative downslope of the *P*-wave (NDAT-P-V8). Iso-V8 was defined as the time from *P*-wave onset to first positive deflection in lead V8. In Cohort 1, a coronary sinus (CS) catheter invasively determined activation timing at the posterolateral LA (mitral isthmus region). In Cohort 2, NDAT-P-V8 and iso-V8 were used to assess the effect of BBP on LA activation. In Cohort 1 (*n* = 50), NDAT-P-V8 strongly correlated with the latest CS activation (*r* = 0.94, *P* < 0.001). In Cohort 2 (*n* = 50), lead V8 facilitated intra-procedural assessment of LA activation. IAB was corrected in 20/23 (87%), accompanied by shortening of NDAT-P-V8 (from 120 ± 25 ms to 82 ± 17 ms, *P* < 0.001) and iso-V8 (from 51 ± 22 ms to 18 ± 17 ms, *P* < 0.001), consistent with earlier LA activation.

**Conclusion:**

NDAT-P-V8 closely reflects the invasively determined timing of the posterolateral LA activation and provides a simple non-invasive marker to assess the effect of BBP on LA activation. Lead V8 may therefore help to assess and optimize atrial activation with BBP.

## Graphical Abstract

**Graphical Abstract:**
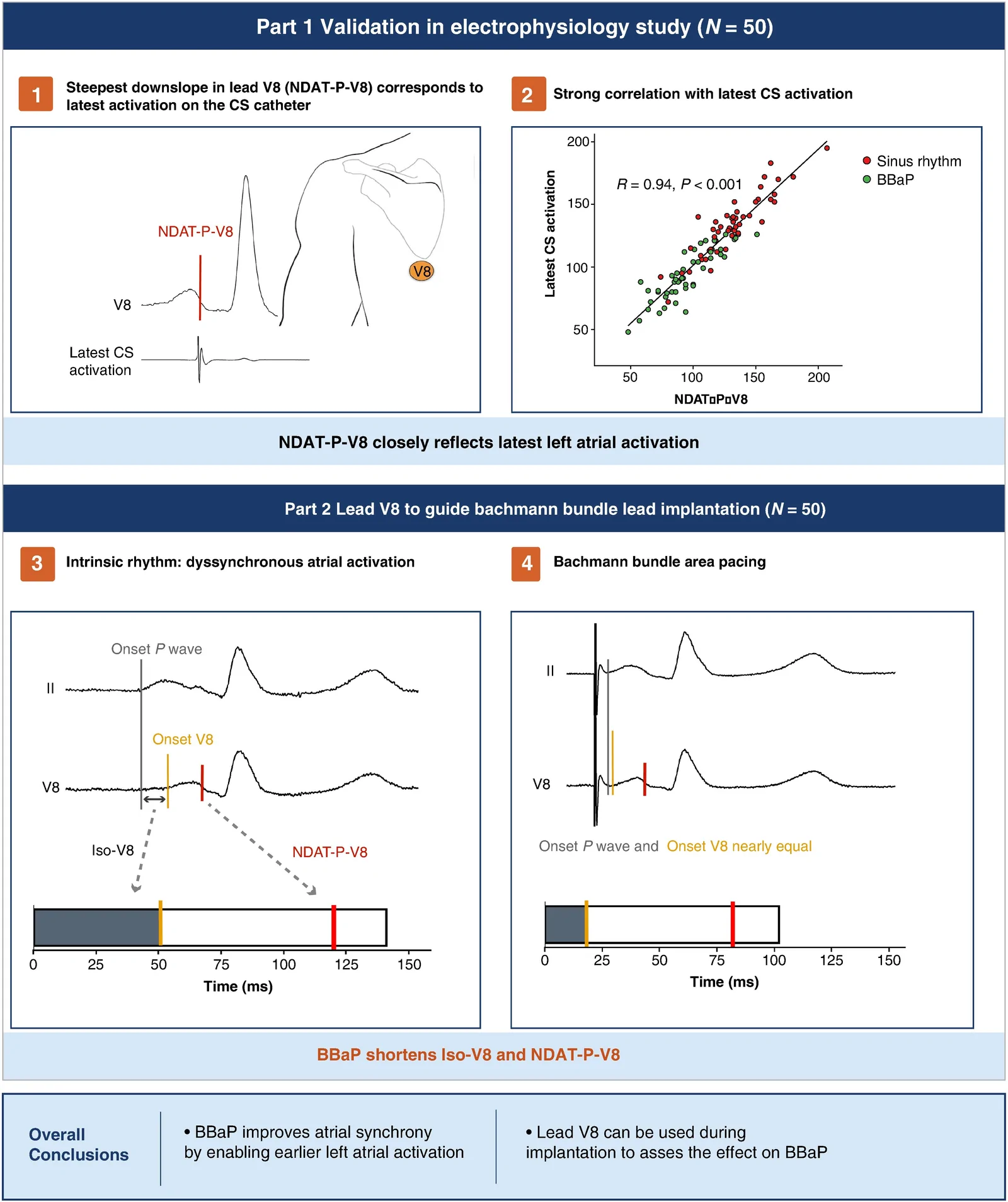
Lead V8 provides a non-invasive surface ECG marker to assess the effect of BBP on LA activation. In the electrophysiology cohort, NDAT-P-V8 closely reflected invasively determined latest LA activation on the CS catheter. In the implantation cohort, representative examples of intrinsic rhythm and BBP illustrate shortening of Iso-V8 and earlier NDAT-P-V8 during BBP, reflecting earlier LA activation. The lower timing bars summarize mean values from the implantation cohort. Lead V5 was repositioned to the V8 location on the back, 2 cm below the inferior angle of the left scapula. BBP, Bachmann bundle pacing; CS, coronary sinus; Iso-V8, isoelectric interval to V8; LA, left atrial; NDAT-P-V8, negative derivative activation time of the *P*-wave in lead V8.

What’s new?

- Repositioning the standard V5 electrode to the posterior V8 position identifies two novel non-invasive surface ECG markers [iso-V8 and the negative derivative of the *P*-wave in V8 (NDAT-P-V8)] that can be used to assess the effect of Bachmann bundle pacing on left atrial activation.
- NDAT-P-V8 demonstrates a strong correlation (*r* = 0.94) with the invasively determined timing of the posterolateral left atrial activation time at the lateral mitral annular region across both intrinsic rhythm and Bachmann bundle pacing.
- Correction of interatrial block during Bachmann bundle pacing was accompanied by shortening of iso-V8 and NDAT-P-V8, supporting the use of lead V8 as a practical intraprocedural tool to assess and optimize atrial activation with Bachmann bundle pacing.

## Introduction

Interatrial conduction delay is a key determinant of atrial synchrony, haemodynamic function, and cardiovascular outcomes.^1^ Delayed left atrial (LA) activation disrupts the coordinated timing of the right and LA contraction, resulting in impaired ventricular filling, elevated LA pressure, and adverse atrial remodelling.^1^ These haemodynamic consequences predispose to atrial fibrillation (AF) and contribute to an increased risk of stroke, heart failure, and death.^2,3^ Conventional right atrial appendage pacing (RAAP) exacerbates interatrial dyssynchrony by prolonging total atrial activation time and producing non-physiological dyssynchronous right-to-left atrial activation.^4^

Bachmann bundle pacing (BBP) promotes interatrial synchrony by targeting the dominant interatrial conduction pathway.^5^ By BBP, the LA activation is advanced, thereby reducing the right-to-left atrial activation delay that underlies interatrial dyssynchrony.^6^ Several criteria have been proposed to identify effective engagement of the Bachmann’s Bundle, integrating fluoroscopic lead positioning with paced *P*-wave morphology, *P*-wave narrowing and axis normalization.^7,8^ However, accurate assessment of *P*-wave duration may be limited by precisely defining *P*-wave offset, as the *P*-wave often returns gradually to the baseline.^9^ Consequently, non-invasive assessment of interatrial synchrony during BBP implantation remains challenging, and objective electrocardiogram (ECG)-based activation criteria are still lacking. Identification of LA activation timing on ECG could therefore be of importance to evaluate the effect of BBP during implantation.

The negative derivative of surface lead V8 (NDAT-V8) has previously been shown to correlate with left ventricular posterolateral wall activation in heart failure patients undergoing cardiac resynchronization therapy with biventricular pacing,^10,11^ as well as during left bundle branch area pacing.^12^ We therefore hypothesized that lead V8 may similarly reflect the timing of the posterolateral LA activation, where atrial activation terminates at the lateral mitral annular region. The negative derivative activation time of the *P*-wave in V8 (NDAT-P-V8) may therefore reflect the local activation of the posterolateral LA region, allowing activation timing to be derived from a surface ECG lead without intracardiac recordings.

Hence, the aim of the present study was to evaluate whether lead V8 can be used to determine the posterolateral LA activation time. Subsequently, we aimed to study whether Lead V8 could be used to assess the effect of BBP on LA activation following lead implantation.

## Methods

### Study design and population

The study included consecutive patients undergoing either an electrophysiology study and catheter ablation (Cohort 1), or BBP lead implantation (Cohort 2). Patients were enrolled between January 2026 and May 2026 at McGill University Health Center (MUHC, Montreal, Canada) and Maastricht University Medical Center (MUMC, Maastricht, the Netherlands). In Cohort 1 of the present study was designed to determine whether NDAT-P-V8 correlates with the latest activation recorded on the coronary sinus catheter. Cohort 2 evaluated the feasibility of using lead V8 to assess the effect of BBP on LA activation, following lead implantation. Surface ECG recordings were obtained using standard EP recording systems CardioLab v8.10, GE Medical at MUHC, and Boston Scientific Lab System PRO EP 4.2.0.25 at MUMC, which enabled 12-lead ECG and filtered and unfiltered electrogram (EGM) recordings (ECG recording: 0.1–500 Hz; filtered EGM: 30Hz-250 Hz). In all patients, the 12-lead ECG was routinely recorded with surface lead V5 positioned at the V8 location, being 2 cm below the inferior angle of the left scapula as shown in *Figure 1*. The study protocol was approved by the respective institutional ethical review boards (METC numbers 19–052 and 2025-0070).

**Figure 1:**
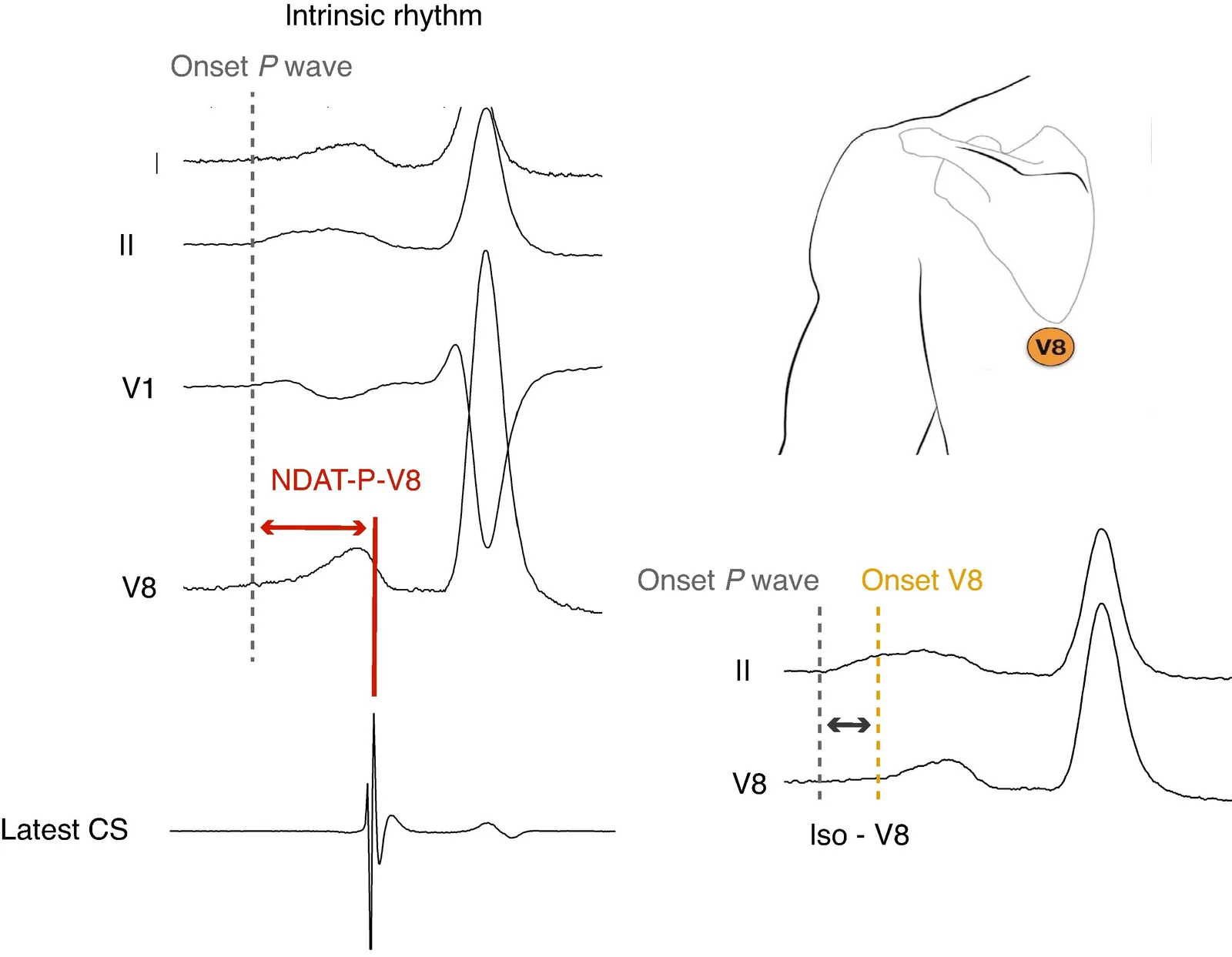
Surface electrode V5 is positioned at the V8 location, 2 cm below the inferior angle of the left scapula. The figure shows leads I, II, V1, and V8 during sinus rhythm. The onset of the P wave is marked by the dashed grey line. NDAT-P-V8 is measured from *P*-wave onset to the steepest negative derivative of the P wave in lead V8, shown by the red line. Iso-V8 is measured from *P*-wave onset to the first positive deflection in lead V8, shown by the dashed orange line. The activation measured in V8 coincides with the latest activation on the coronary sinus catheter. CS, coronary sinus; Iso-V8, isoelectric interval to V8; NDAT-P-V8, negative derivative activation time of the *P*-wave in lead V8.

### Cohort 1: temporary BBP during invasive electrophysiology study and ablation

Patients undergoing catheter ablation were included if a coronary sinus (CS) catheter was present and its tip was able to be advanced to the lateral mitral annular region to map the posterolateral LA activation time. The posterolateral LA activation time was determined from bipolar electrograms recorded from the CS catheter and defined as the latest local activation recorded across all CS bipoles, with local activation time identified at the peak of the bipolar deflection. Temporary pacing was then performed at the Bachmann bundle within the right atrium with a diagnostic or ablation catheter. Pacing was performed at an output of 5–10 mA, a pulse width of 2 ms, and at a rate ∼10 bpm faster than the baseline intrinsic rate. BBP was confirmed with identification of a multiphasic electrogram at the inferoseptal aspect of the superior vena cava, with pace-mapping performed to produce a positive, peaked *P*-wave in the inferior leads with normalization of the axis and shortening of the *P*-wave duration.^7^

### Cohort 2: BBP lead implantation

Consecutive patients undergoing a transvenous BBP lead implantation were included. BBP was performed using a Model 3830 SelectSecure lead (Medtronic, Minneapolis) advanced through a dedicated sheath (Medtronic C315-S4 or C315-S5). The lead was implanted at the Bachmann bundle as previously described.^7^ In short, a multiphasic component on the filtered EGM, starting after the onset of the surface *P*-wave, was identified with the tip of the lead positioned poster-medial at the inferoseptal site of the superior vena cava. Pace-mapping was performed to identify an optimal position prior to deployment, as noted by symmetrically positive and peaked *P*-waves in the inferior leads with axis normalization, and shortening of the paced *P*-wave duration.^7^ The lead was then deployed with several rotations of the lead, with clear current of injury on the unfiltered electrogram derived from the tip of the lead ensuring adequate tissue contact. After withdrawal and slitting of the sheath, measurements were obtained at an atrial pacing rate ∼10 bpm faster than the intrinsic rate.

### ECG measurements and definitions

The following measurements were obtained in both Cohort 1 and Cohort 2 during intrinsic rhythm and BBP: *P*-wave duration, stimulus-to-*P*-wave onset, PR interval, iso-electric interval to V8 (iso-V8), and NDAT-P-V8. *P*-wave duration was defined as the interval from the onset of the earliest *P*-wave (usually lead II) to the latest end of the *P* wave on any surface ECG lead. In lead V8, the onset of the *P* wave occurs later than in the standard 12-lead ECG leads, resulting in an apparent isoelectric interval. Iso-V8 was defined as the interval from *P*-wave onset to the beginning of the steep positive upslope of the *P* wave in lead V8 (*Figure 1*). Iso-V8 was considered an exploratory marker that may reflect the delay to LA activation via conduction over Bachmann's bundle. NDAT-P-V8 was defined as the interval from the earliest *P*-wave onset (usually lead II) to the steepest negative downslope of the *P* wave in lead V8. Finally, interatrial block (IAB) was defined based on *P*-wave morphology and duration. Partial IAB was defined as a *P*-wave duration >120 ms with a bimodal *P* wave in leads II, III, or aVF, whereas advanced IAB was defined as a *P*-wave duration >120 ms with biphasic and negative terminal component in leads II, III, and aVF. The interval from *P*-wave onset to the latest coronary sinus activation was recorded in Cohort 1. Interobserver variability was assessed in 15 randomly selected cases, independently measured by two investigators (S.M. and A.F.) for NDAT-P-V8, iso-V8 and latest CS activation.

### Statistical analysis

Continuous variables are presented as mean ± standard deviation or median with interquartile range (IQR) based on their distribution. To evaluate the correlation between the NDAT-P-V8 and the latest coronary sinus activation on the CS lead, linear regression analysis was performed, and the Pearson’s correlation coefficient (r) was calculated. Agreement between NDAT-P-V8 and the distal CS activation was further evaluated using Bland–Altman analysis, with results reported as mean bias ± limits of agreement. Comparisons between intrinsic rhythm and BBP were performed using paired *t*-tests or Wilcoxon signed-rank tests. For these analyses, normality was assessed by visual inspection of histograms and Q-Q plots. All analyses were performed using SPSS version 31.0 and RStudio version 2026.01.0 + 392.

## Results

### Patient characteristics

In Cohort 1 of the study, a total of 50 patients undergoing temporary BBP during catheter ablation procedures were included (*Table 1*, Left column). The mean age was 64 ± 10 years, and 31 patients (62%) were male. The mean left ventricular ejection fraction (LVEF) was 53 ± 9% and mean LA volume index (LAVI) was 40 ± 12 mL/m^2^. Nine patients (18%) had undergone a prior AF ablation. The intrinsic heart rate was 66 ± 18 bpm.

**Table 1:** Baseline patient characteristics of the patients undergoing temporary BBP during catheter ablation (left column), and those undergoing permanent BBP lead implantation (right column)

	Temporary BBP (*n* = 50)	BBP lead implantation (*n* = 50)
Age (years), mean ± SD	64 ± 10	72 ± 12
Male sex, *n* (%)	31 (62)	35 (70)
BMI (kg/m^2^), mean ± SD	30 ± 5	26 ± 4
Intrinsic heart rate	66 ± 18	62 ± 15
**Medical history**		
Hypertension, *n* (%)	26 (52)	32 (64)
Diabetes Mellitus, *n* (%)	7 (14)	18 (36)
Prior AF ablation, *n* (%)	9 (18)	2 (4)
**Echocardiography**		
LVEF (%), mean ± SD	53 ± 9	47 ± 15
LAVI (mL/m^2^), mean ± SD	40 ± 12	39 ± 10
**Indication for device implantation**		
AV block, *n* (%)	—	11 (22)
SND, *n* (%)	—	19 (38)
CRT, *n* (%)	—	11 (22)
Other, *n* (%)	—	9 (18)

Abbreviations: AF, atrial fibrillation; AV, atrioventricular; BBP, Bachmann bundle pacing; BMI, body mass index; CRT, cardiac resynchronization therapy; LAVI, left atrial volume index; LVEF, left ventricular ejection fraction; SND, sinus node dysfunction.

For BBP lead implantation measurements in Cohort 2, BBP lead implantation was performed in 50 patients (*Table 1*, column 2). The indications for pacemaker were sinus node dysfunction in 38% (19/50), atrio-ventricular block in 22% (11/50), and cardiac resynchronization in 22% (11/50). The mean age was 73 ± 11, 65% was male, with an LVEF of 47 ± 15%, and a LAVI of 39 ± 10 mL/m^2^. The intrinsic heart rate was 62 ± 15 bpm.

### Cohort 1: temporary BBP

#### Validation of NDAT-P-V8 as a marker of posterolateral LA activation time

There was a very strong correlation between the NDAT-P-V8 and the latest activation on the CS catheter (*r* = 0.94, *p* < 0.001; *Figure 2A*). This correlation was strong during both temporary BBP (*r* = 0.89, *P* < 0.001) and during intrinsic rhythm (*r* = 0.91, *P* < 0.001). Bland–Altman analysis showed a mean bias of 0.2 ms (SD 11), with 95% limits of agreement from −21 to 22 ms (*Figure 2B*). Interobserver agreement was high, with a mean bias of 1 ± 8 ms for NDAT-P-V8 and −1 ± 8 ms for latest CS activation, whereas interobserver agreement for iso-V8 was lower, with a mean difference of 6 ± 16 ms.

**Figure 2:**
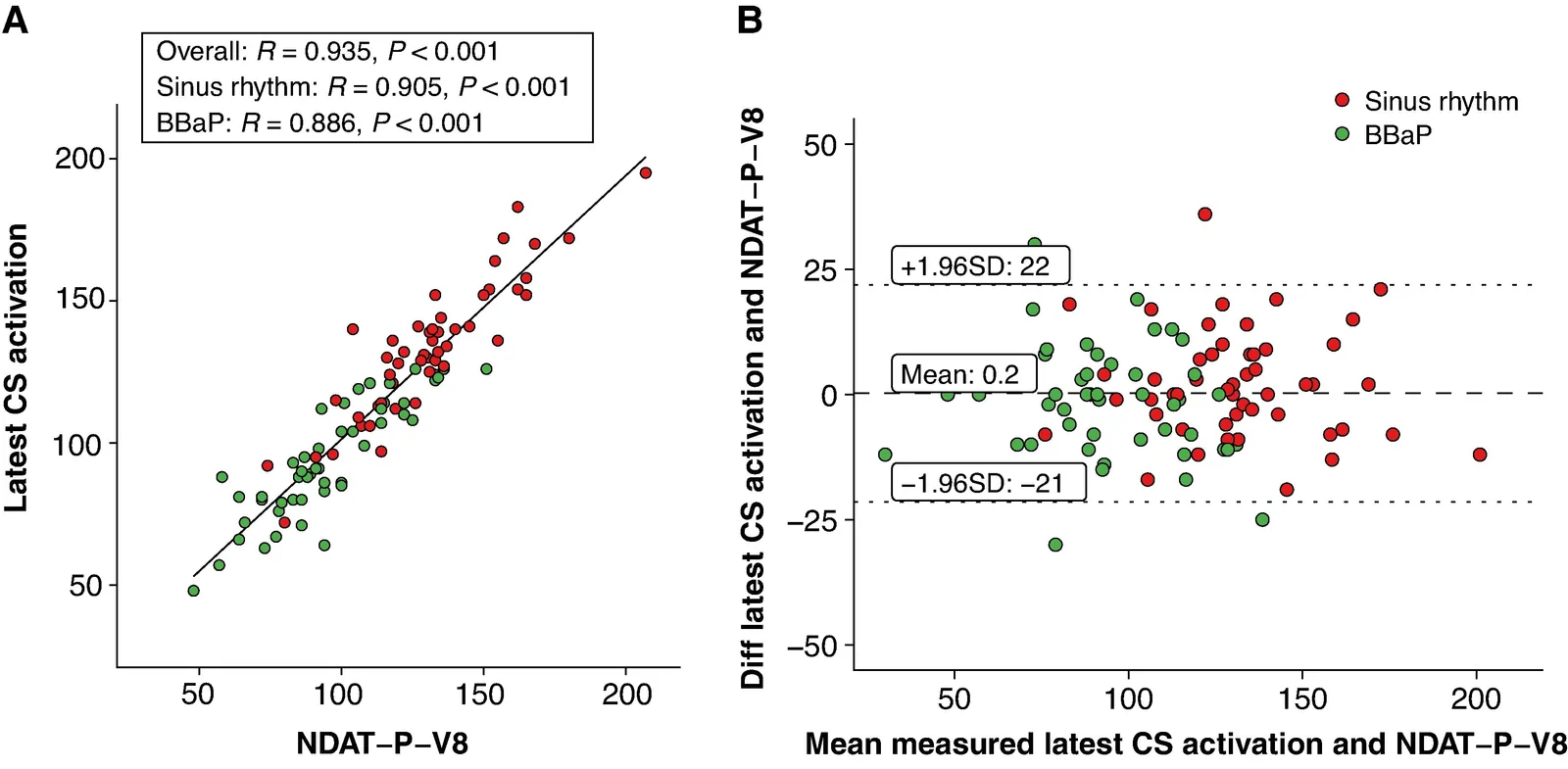
(*A*) Scatter plot showing the correlation between NDAT-P-V8 and latest activation on the coronary sinus catheter. (*B*) Bland–Altman plot showing the agreement between NDAT-P-V8 and latest activation on the coronary sinus catheter. BBP, Bachmann bundle pacing; CS, coronary sinus; NDAT-P-V8, negative derivative activation time of the *P*-wave in lead V8.

To further illustrate NDAT-P-V8 and its relation with the posterolateral LA activation, *Figure 3* provides a representative electroanatomic mapping example. The three-dimensional electroanatomic LA map demonstrates that the latest site of activation is located at the lateral mitral annular region, anterior and caudal to the left inferior pulmonary vein (LIPV). The latest signal on the activation map coincides with both the latest CS signal and the NDAT-P-V8 on the surface ECG.

**Figure 3:**
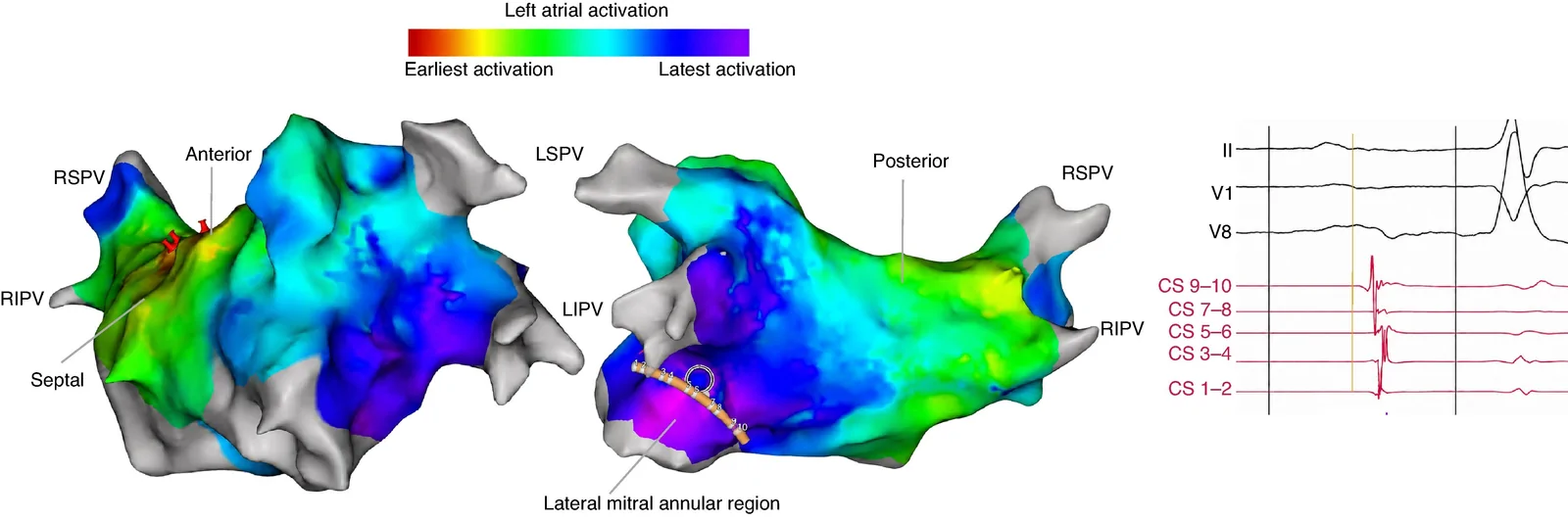
3-dimensional electroanatomic map of the activation of the left atrium. The latest activation (purple) is noted at the inferior Marshall ridge, anterior and caudal to the LIPV. The latest activation is represented on the mapping catheter (Octaray, Biosense, Johnson & Johnson), and coincides with both the latest CS signal and the NDAT-P-V8. LAT, local activation time; LIPV, left inferior pulmonary vein; LSPV, left superior pulmonary vein; RIPV, right inferior pulmonary vein; RSPV, right superior pulmonary vein.

#### P-wave changes

The *P*-wave duration was shorter during temporary BBP compared with intrinsic rhythm (151 ± 26 ms vs. 113 ± 22 ms) as shown in *Table 2*. BBP reduced the iso-V8 interval as compared to intrinsic (52 ± 22 ms vs. 30 ± 19 ms, *P* < 0.001), and NDAT-P-V8 was similarly reduced during BBP as compared to intrinsic rhythm (130 ± 24 ms vs. 94 ± 22 ms, *P* < 0.001). The latest CS activation time was shorter during BBP than during intrinsic rhythm (92 ± 22 ms vs. 133 ± 23 ms). An example of a patient with partial IAB, characterized by a broad and notched *P* wave in lead II, is shown in *Figure 4*. In this example, a prolonged apparent isoelectric interval was observed before the steep positive upslope of the *P* wave in lead V8, which may be consistent with delayed right-to-left atrial activation and thus interatrial dyssynchrony. During temporary BBP, correction of partial IAB was accompanied by shortening of NDAT-P-V8 from 162 to 94 ms and iso-V8 from 101 to 25 ms. The reduction in NDAT-P-V8 was consistent with earlier posterolateral LA activation.

**Figure 4:**
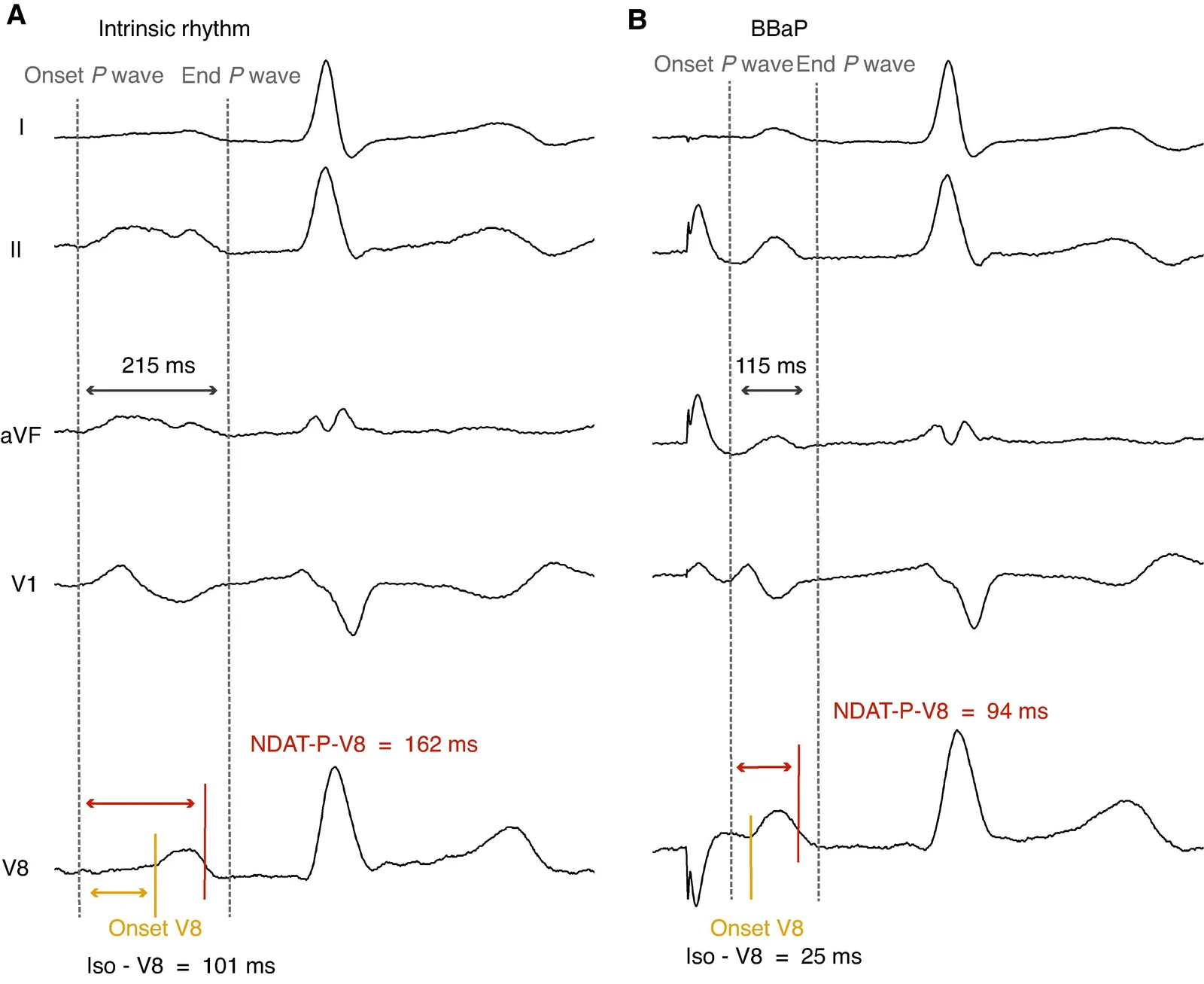
(*A*) Intrinsic rhythm and (*B*) pacing at the bachmann bundle with a focal ablation catheter. During temporary BBP, correction of partial IAB was accompanied by shortening of NDAT-P-V8 from 162 to 94 ms and iso-V8 from 101 to 25 ms. BBP, Bachmann bundle pacing; Iso-V8, isoelectric interval to V8; NDAT-P-V8, negative derivative activation time of the *P*-wave in lead V8.

**Table 2:** *P*-wave measurements during electrophysiology study and ablation

	Intrinsic rhythm (*n* = 50)	Temporary BBP (*n* = 50)	*P*-value^a^
*P*-wave duration (ms)	151 ± 26	113 ± 22	<0.001
V8 *P*-wave duration (ms)	99 ± 22	82 ± 18	<0.001
NDAT-P-V8	130 ± 25	94 ± 24	<0.001
Latest CS activation (ms)	133 ± 24	92 ± 22	<0.001
Iso-V8 (ms)	52 ± 23	30 ± 19	<0.001
PR interval (ms)	191 ± 39	163 ± 47	<0.001

Values are presented as mean ± standard deviation.

^a^Paired *t*-test.

Abbreviations: CS, coronary sinus; Iso-V8, isoelectric interval to V8; NDAT-P-V8, negative derivative activation time of the *P*-wave in lead V8.

### Cohort 2: BBP lead implantation

BBP lead implantation was performed in 50 consecutive patients. All BBP lead implantations were performed during sinus rhythm. Baseline patient characteristics are described in *Table 1*, right column. Evaluation of NDAT-P-V8 and Iso-V8 was feasible in all BBP implantation procedures. *P*-wave measurements obtained during implantation are shown in *Table 3*. Compared with intrinsic rhythm, the total *P*-wave duration was shorter during BBP from 141 ± 24 ms to 102 ± 16 ms, and NDAT-P-V8 was reduced from 120 ± 25 ms to 82 ± 17 ms (*P* < 0.001). This reduction is probably related with earlier LA activation, as reflected on the ECG by a shortening of the iso-V8 from 51 ± 22 ms to 18 ± 17 ms (*P* < 0.001). The PR interval also shortened from 211 [IQR 172–252] ms during intrinsic rhythm to 170 [IQR 136–208] ms during BBP while pacing at 10 bpm faster rate than the intrinsic rate. Partial IAB was present in 23 cases (46%) which could be corrected in 20 (87%) with BBP (*P* = 0.001).

**Table 3:** *P*-wave measurements during BBP lead and pacemaker implantation

	Intrinsic rhythm (*n* = 50)	BBP (*n* = 50)	*P*-value
*P*-wave duration (ms)	141 ± 24	102 ± 16	< 0.001^a^
Stim to onset *P* wave (ms)	—	23 ± 20	—
Iso-V8 interval (ms)	51 ± 22	18 ± 17	< 0.001^a^
NDAT-*P*-V8 (ms)	120 ± 25	82 ± 17	< 0.001^a^
Partial interatrial block, *n* (%)	23 (46)	3 (6)	0.001^b^
Advanced interatrial block, *n* (%)	4 (8)	1 (2)	0.125^b^
PR (interval; ms)	211 (172–252)	170 (136–208)	0.001^c^

Values are presented as mean ± standard deviation or median with interquartile range, and categorical as numbers with percentages.

^a^ *P*-value calculated using a paired *t*-test.

^b^ *P*-value calculated using McNemar’s test.

^c^ *P*-value calculated using Wilcoxon signed-rank tests

Abbreviations: BBP, Bachmann bundle pacing; Iso-V8, isoelectric interval to V8; NDAT-*P*-V8, negative derivative activation time of the *P*-wave in lead V8.

A typical example of correction of the partial IAB is provided in *Figure 5*. During BBP, a 41 ms shortening of the NDAT-P-V8 was observed, consistent with a reduction posterolateral LA activation time, and more synchronous atrial activation. *Figure 6* demonstrates 2 further examples of correction (*Figure 6A*) and persistent IAB with BBP (*Figure 6B*).

**Figure 5:**
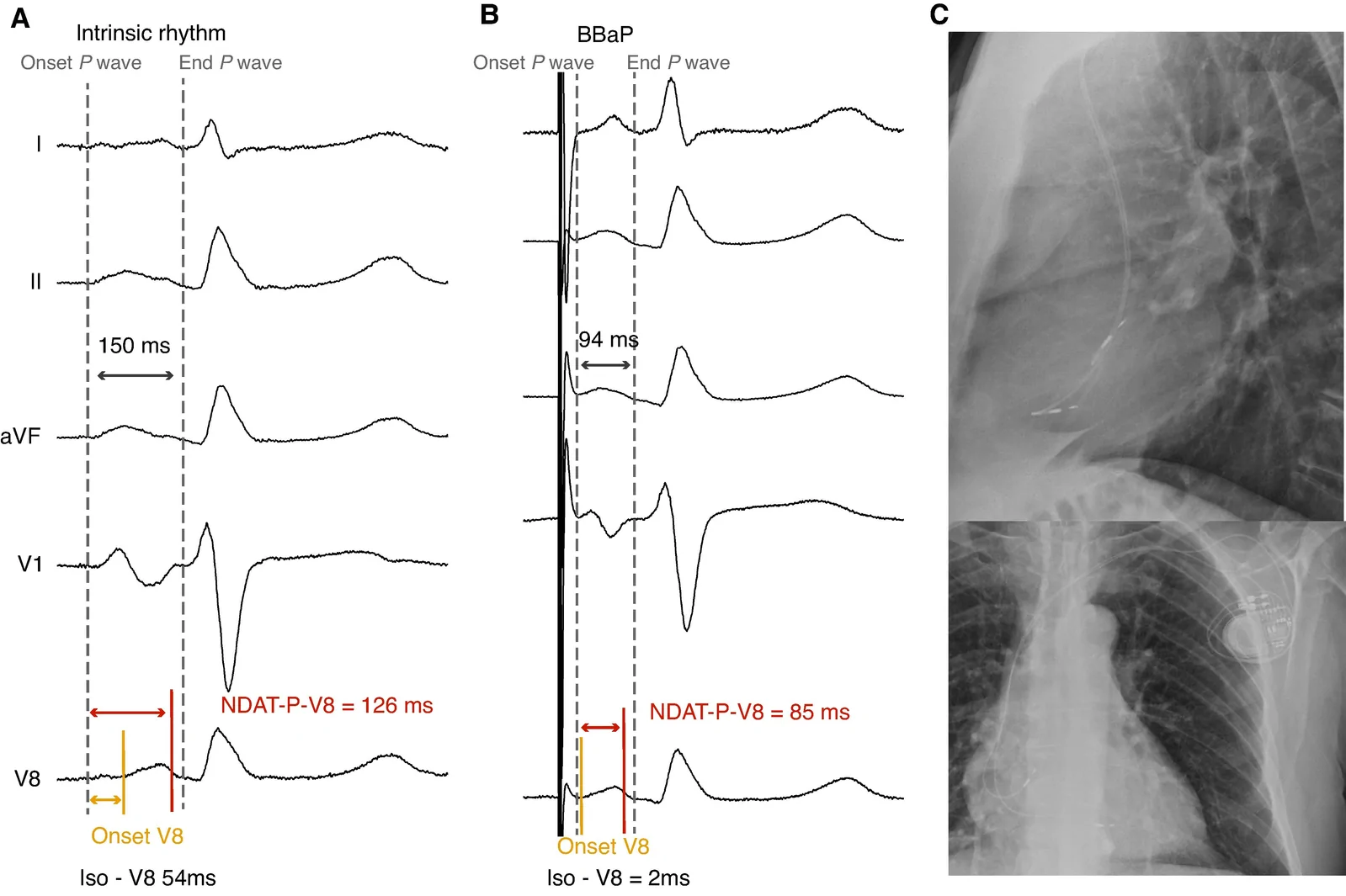
(*A*) Intrinsic rhythm, (*B*) bachmann bundle pacing, and (*C*) post-implantation chest radiograph, illustrating the use of *P*-wave NDAT-V8 and the iso-V8 interval during BB lead implantation. BBP, Bachmann bundle pacing; Iso-V8, isoelectric interval to V8; NDAT-P-V8, negative derivative activation time of the *P*-wave in lead V8.

**Figure 6:**
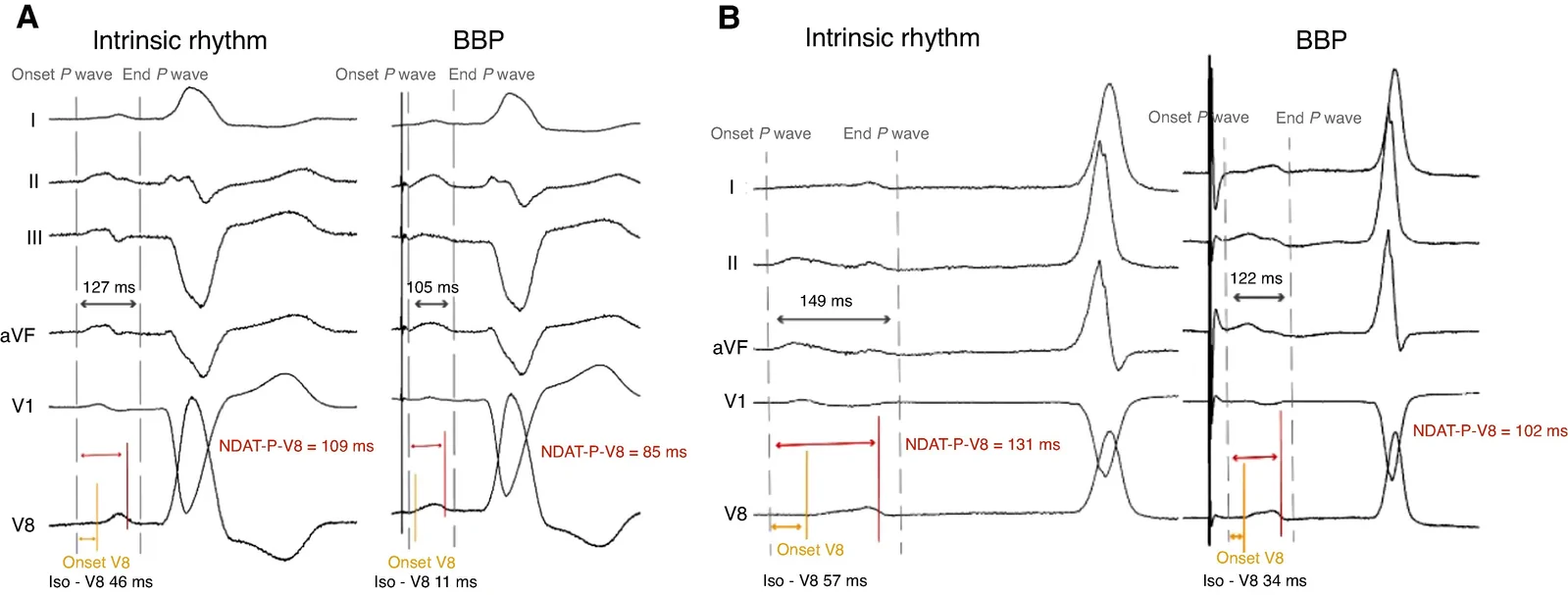
The intrinsic P wave and Bachmann pacing are seen in two separate patients where in (*A*) correction of the baseline interatrial block has been achieved with BBP; and (*B*) there remains persistent interatrial block with BBP. BBP, Bachmann bundle pacing; Iso-V8, isoelectric interval to V8; NDAT-P-V8, negative derivative activation time of the *P*-wave in lead V8.

## Discussion

This study demonstrates that the *P*-wave obtained from lead V8 can guide the implanting physician to determine the degree of atrial synchrony obtained during BBP implantation. The main findings of the current study are as follows: (i) The NDAT-P-V8 closely reflects the posterolateral LA activation time at the lateral mitral annular region with a strong correlation with the invasively-measured latest activation on the CS catheter; (ii) The NDAT-P-V8 was considerably shorter during temporary BBP as compared to intrinsic rhythm, indicating earlier posterolateral LA activation time; and (iii) During BBP pacemaker implantation, NDAT-P-V8 and Iso-V8 were readily measurable on surface ECG, supporting the feasibility of implementing lead V8 in daily implantation practice to guide the implanting physician in determining the extent of advancement of LA activation during BBP.

### NDAT-P-V8 as a non-invasive marker of posterolateral left atrial activation

The electrical activation of the LA normally occurs through several muscular connections linking the right and LA.^13^ The dominant interatrial pathway, however, is Bachmann’s bundle, a broad band of directionally oriented atrial myocardial fibres that extend from the sinus node and traverse the interatrial groove toward the roof and anterior LA.^14^ Extensions of Bachmann’s bundle pass anteriorly to encircle the LA appendage and then blend in with the lateral atrial wall, or pass superiorly to the left lateral ridge to pass in front of the left pulmonary vein ostia before blending into the musculature of the inferior wall.^15^ Secondary and slower interatrial connections pass through connections at the coronary sinus as well as the interatrial septum. Consequently, the latest activation in the LA is at the inferior Marshall ridge, anterior and caudal to the LIPV. As the coronary sinus catheter can be positioned around the mitral annulus, this latest activation can therefore be mapped by CS recordings.

Non-invasive assessment of the latest LA activation on the surface ECG remains challenging. However, activation timing can be derived from unipolar surface ECG recordings, which capture the passage of the activation wavefront, producing a positive deflection as the wavefront approaches the electrode, and a negative deflection as it passes away. The point of maximal negative slope, referred to as negative derivative activation time (NDAT), therefore reflects the moment the wavefront passes beneath the electrode and represents the local activation on the surface ECG.^16^ As lead V8 is positioned posteriorly, the NDAT of the *P*-wave provides a non-invasive estimate of the timing of latest LA activation.

The present study confirms this concept by showing that NDAT-P-V8 correlates strongly with the latest atrial activation recorded on the coronary sinus catheter during electrophysiology study, validating its use as a non-invasive marker of LA activation time. A similar concept has previously been validated by our group for the latest left ventricular activation during wide QRS (left bundle branch block and right bundle branch block), where the NDAT of the QRS in lead V8 correlated closely with the QLV, an invasive marker of delayed left ventricular posterolateral wall activation.^10,11^ In addition, this correlation was also present during LBBAP where V8 can potentially be used to guide the resynchronization effect.^12^

### Lead V8 to assess the effect of BBP on left atrial activation

Assessment of *P*-wave duration during BBP may be complicated by variability in isoelectric line and gradual return of the *P*-wave to baseline.^9^ NDAT-P-V8 allows a more reproducible measurement during both intrinsic rhythm and atrial pacing, as it is based on the steepest negative downslope of the *P*-wave in lead V8, reflected by the low interobserver variability in our study. Iso-V8 showed lower interobserver reproducibility, likely reflecting the difficulty of identifying the onset of a gradual positive deflection in lead V8 in some patients. In the present study, iso-V8 was therefore considered a secondary, exploratory parameter, and its precise physiological interpretation requires further validation. Based on the hypothesis that the positive *P*-wave deflection in lead V8 may be related to the onset of LA activation, a prolonged iso-V8 during intrinsic rhythm may reflect impaired interatrial conduction over Bachmann’s bundle and delayed right-to-left atrial activation, whereas shortening of iso-V8 during BBP may suggest earlier LA activation and improved interatrial synchrony. However, larger prospective studies are needed to determine whether a specific degree of NDAT-P-V8 or iso-V8 shortening can be established as a threshold for adequate atrial synchrony during BBP.

### Interatrial resynchronization during BBP

Interatrial block on ECG is characterized by a prolonged *P*-wave duration (≥120 ms) with notched or bimodal morphology in the inferior leads (partial block), or by a terminal negative deflection in the inferior leads (advanced block), reflecting caudo-cranial activation via the septal or coronary sinus interatrial connections.^2^ Advanced interatrial block is associated with an increased risk of AF and stroke, and worse prognosis in patients with HF with preserved ejection fraction, emphasizing the importance of LA electrical synchrony for optimal function.^14,17–19^ This is particularly relevant in our BBP lead implantation cohort, as partial IAB, was present in 46% of patients, and successfully corrected in 87% of these cases. As shown in *Figure 4*, correction of partial IAB was accompanied by marked shortening of both NDAT-P-V8 and Iso-V8.

### Atrioventricular synchrony

BBP may simultaneously improve interatrial synchrony and shorten AV timing, thereby addressing both atrial dyssynchrony and suboptimal AV coupling. In our study, BBP was associated with significant shortening of the PR interval during pacing at 10 bpm faster than intrinsic heart rate in both study cohorts. This is likely related to the electrophysiological properties of Bachmann’s Bundle, where conduction velocities are high, facilitating preferential interatrial conduction toward the AV node.^20^ Activation in the LA may reach the AV node through a distinct left-sided input, related to the mitral annulus.^21^ Accordingly, shortening of Iso-V8 during BBP may be particularly relevant, as it may indicate earlier LA activation with earlier engagement of left-sided atrioventricular inputs and can thereby serve as a useful marker of earlier AV nodal activation. This may be clinically relevant, as prolonged AV delays have been associated with increased end-diastolic pressure and impaired cardiac function.^22^

### Limitations

Several limitations are of consideration in the present study. First, LA activation patterns may be altered in patients who underwent previous AF ablation procedures, which might have influenced the activation over Bachmann’s bundle. However, only a small proportion of our patients underwent redo ablation (18%), and the strong correlation was observed even in this population. Next, incomplete distal advancement of the CS catheter in some patients may have led to underestimation of the latest LA activation time. Finally, Cohort 1 of our study, the temporary BBP cohort, showed longer *P*-wave durations and NDAT-P-V8 compared with the BBP implantation cohort, likely due to atrial remodelling in these patients undergoing AF ablation limiting direct comparison between cohorts. Despite these differences, both cohorts demonstrated consistent shortening of NDAT-P-V8 and iso-V8, supporting the use of lead V8 to assess the effect of BBP on LA activation across different patient populations.

## Conclusion

The negative derivative of the *P*-wave measured in ECG lead V8 (NDAT-P-V8) closely reflects the invasively derived timing of the posterolateral LA activation, thereby providing a non-invasive electrocardiographic surrogate. By offering simple and reproducible measurements of posterolateral LA activation timing, reflected in the NDAT-P-V8, lead V8 may facilitate assessment of BBP and help optimize LA activation. These results support feasibility of implementing lead V8 in daily practice to guide the implanting physician in assessing and optimizing LA activation with BBP.

## Data Availability

The data underlying this article will be shared on reasonable request to the corresponding author.
